# *Bacteroides vulgatus* alleviates dextran sodium sulfate-induced colitis and depression-like behaviour by facilitating gut-brain axis balance

**DOI:** 10.3389/fmicb.2023.1287271

**Published:** 2023-11-16

**Authors:** Xing Wu, Jiahao Xu, Jingbo Li, Minzi Deng, Zhaohua Shen, Kai Nie, Weiwei Luo, Chao Zhang, Kejia Ma, Xuejie Chen, Xiaoyan Wang

**Affiliations:** ^1^Department of Gastroenterology, The Third Xiangya Hospital, Central South University, Changsha, China; ^2^Hunan Key Laboratory of Nonresolving Inflammation and Cancer, Cancer Research Institute, Central South University, Changsha, China; ^3^Furong Laboratory, Changsha, Hunan, China

**Keywords:** *Bacteroides vulgatus*, inflammatory bowel disease, depression, gut–brain axis, p-hydroxyphenylacetic acid

## Abstract

**Background:**

Patients with inflammatory bowel disease (IBD) have a higher prevalence of depression. Gut microbiota dysbiosis plays an important role in IBD and depression. However, few studies have explored the characteristic microbiota of patients with IBD and depression (IBDD), or their role in IBDD.

**Methods:**

We performed deep metagenomic sequencing and 16S rDNA quantitative PCR to characterise the gut microbial communities of patients with IBDD and patients with IBD without depression (IBDND). We then assessed the effect of the microbiota on colitis and depression in mouse models of dextran sulfate sodium salt (DSS)-induced colitis and lipopolysaccharide (LPS)-induced depression. Furthermore, liquid chromatography–tandem mass spectrometry was used to analyse the microbiota-derived metabolites involved in gut–brain communication. Evans Blue tracer dye was used to assess blood–brain barrier (BBB) permeability.

**Results:**

Our results showed that the faecal abundance of *Bacteroides vulgatus* (*B. vulgatus*) was lower in patients with IBDD than in those with IBDND. In the DSS-induced colitis mouse model, the *B. vulgatus* group showed a significantly lower disease activity index score, lesser weight loss, and longer colon length than the DSS group. Moreover, *B. vulgatus* relieved depression-like behaviour in the DSS-induced colitis mouse model and in the LPS-induced depression mouse model. Furthermore, the key metabolite of *B. vulgatus* was p-hydroxyphenylacetic acid (4-HPAA), which was found to relieve intestinal inflammation and alleviate depression-like behaviours in mouse models. By increasing the expression of the tight junction protein claudin-5 in the vascular endothelium of the BBB, *B. vulgatus* and 4-HPAA play critical roles in gut–brain communication.

**Conclusion:**

*B. vulgatus* and *B. vulgatus*-derived 4-HPAA ameliorated intestinal inflammation and relieved depressive symptoms through the gut–brain axis. Thus, administration of *B. vulgatus* or 4-HPAA supplementation is a promising therapeutic strategy for treating IBD, particularly IBDD.

## Introduction

The incidence of inflammatory bowel disease (IBD), including ulcerative colitis (UC) and Crohn’s disease (CD), has increased in the past few years (Kaplan and Windsor, 2021). The occurrence and development of IBD are closely related to a variety of factors, including microbiota, environment, genes, and psychology (Ananthakrishnan, 2015; Borren et al., 2019). Depression is commonly accompanied by IBD, with a prevalence of 25.2% (Barberio et al., 2021). Patients with IBD who experience depression are more likely to relapse, undergo surgery, or be hospitalised (Kochar et al., 2018). Antidepressant treatment protects against IBD (Goodhand et al., 2012; Frolkis et al., 2019). However, a safe and effective regimen that simultaneously treats intestinal inflammation and depression has not been fully developed (Mikocka-Walus et al., 2020). Therefore, the discovery of therapeutic strategies for the treatment of patients with IBD, especially those with depression, is important.

A large body of evidence suggests that IBD is associated with a reduction in the number of key commensal gut bacteria (Lee and Chang, 2021). Increasing evidence has indicated that some commensal gut bacteria possess antidepressant properties (Simpson et al., 2021). A clinical trial showed that *Bifidobacterium longum* reduced depression scores and altered brain activity in patients (Pinto-Sanchez et al., 2017). Additionally, in clinical studies, *Lactobacillus plantarum* and *Bifidobacterium breve* modulated the gut microbiota or metabolism to reduce the symptoms of major depressive disorders (Rudzki et al., 2019; Tian et al., 2022). Nevertheless, few studies have explored the characteristic microbiota of patients with IBD and depression (IBDD) and their role in IBDD.

The gut–brain axis is a network of connections between gut bacteria and the brain that allows bidirectional communication via several communication pathways, including chemical transmitters, neuronal pathways, and the immune system (Morais et al., 2021). For instance, in mice, the bacterial metabolite 4-ethylphenyl sulfate enters the brain, impairs oligodendrocyte maturation, decreases oligodendrocyte–neuron interactions, and induces anxiety-like behaviour (Needham et al., 2022). In addition, the administration of microbiota-derived short-chain fatty acids to mice alters the expression of brain-derived neurotrophic factor (BDNF), a neuronal factor associated with depression (Han et al., 2014). Additionally, methylamine trimethylamine N-oxide, which is associated with the microbiome, enhances the integrity of the blood–brain barrier (BBB) and protects murine cognitive function (Hoyles et al., 2021). However, it is still unclear how enterobacteria regulate the gut–brain axis function and depressive symptoms in IBDD.

In this study, we performed deep metagenomic sequencing and 16S rDNA quantitative PCR (qPCR) to characterise the gut microbial communities in patients with IBDD and IBD without depression (IBDND). We found a significant difference in the abundance of *Bacteroides vulgatus* (*B. vulgatus*) between the two groups. We assessed the effect of *B. vulgatus* on colitis and depression in mouse models of dextran sulfate sodium salt (DSS)-induced colitis and lipopolysaccharide (LPS)-induced depression. Furthermore, liquid chromatography–tandem mass spectrometry (LC–MS/MS) was used to analyse *B. vulgatus* metabolites involved in gut–brain communication.

## Materials and methods

### Participants and sample collection

The participants were recruited from the Third Xiangya Hospital of Central South University. This study was approved by the Institutional Review Board of the Third Xiangya Hospital of Central South University and written informed consent was obtained from all participants. Diagnosis of IBD was confirmed using previously established international criteria based on clinical, endoscopic, histopathological, and radiological findings (Gomollon et al., 2017; Magro et al., 2017). The Patient Health Questionnaire-9 (PHQ-9) was used to evaluate depression levels, and PHQ-9 scores >4 points were defined as depression (Spitzer et al., 1999). Stool samples were collected from participants if antibacterial medications had not been administered within 3 months. First, we collected faecal samples from six patients with CD with depression (CDD) and from 16 patients with CD without depression (CDND) for metagenomic sequencing. Furthermore, additional faecal samples were collected from 25 patients with CDD, 24 with CDND, 10 with UC with depression (UCD), and 5 without depression (UCND), and from 40 healthy controls (HCs); these patients were considered an expanded cohort for the qPCR experiment. No significant differences were found in the general information of metagenome sequencing or expanded cohorts (Supplementary Tables S1, S2).

### Metagenomic sequencing and analysis

Faecal samples were collected from all participants as described in a previous study and sequenced on an Illumina HiSeq platform (Jian et al., 2020). Low-quality sequence reads were discarded from raw sequencing data, and human host DNA was eliminated by filtering out the human reference genome (hg38). Clean data were assembled using SOAPdenovo software (version 2.04), and taxonomic analyses were conducted using Kraken (version 1.0). The values of species abundance in metagenome sequencing results are listed in Supplementary Table S3.

To estimate bacterial diversity (within and between samples) and dissimilarity (between samples), the Shannon index and Bray–Curtis dissimilarity metric were estimated using R (version 3.5.0) and the vegan package (version 2.5–2). Partial least squares discriminant analysis (PLS-DA) was used to reveal the taxonomic changes between the two groups. The Bray–Curtis distance was used to assess the significance of differences between the two groups using permutational multivariate analysis. The Linear discriminant analysis (LDA) Effect Size (LEfSe) method was used to analyse differential abundances. A value of *p* threshold of 0.05 (Wilcoxon rank sum test) was considered statistically significant.

### 16S ribosomal DNA qPCR

Bacterial DNA was extracted from human or mouse faecal samples using a Stool Genomic DNA Kit (TransGen Biotech, China). Real-time qPCR was performed using a Bio-Rad CFX96 Real-Time PCR platform. The reaction mixture (20 μL) for qPCR contained SYBR qPCR Master Mix (Vazyme, China) (10 μL), forward and reverse primers (final concentration 200 nM), and the extracted DNA (4 μL). The relative abundance was calculated using the ΔCt method and normalised to the total bacterial count. The following primer sets were used: *B. vulgatus*: 5′-ACTGGATTTGACAGCGTGGC-3′ and 5′-AAACGCTTCGGAACAAGAGCT-3′; total bacteria (Atarashi et al., 2011): 5′-GGTGAATACGTTCCCGG-3′ and 5′-TACGGCTA CCTTGTTACGACTT-3′.

### GMrepo database analysis

The relative abundances of *B. vulgatus* in stool samples from patients with IBD, patients with depression, and HC were extracted and analysed using the GMrepo database^1^ (Wu et al., 2020).

### Preparation of *Bacteroides vulgatus*

*Bacteroides vulgatus* ATCC 8482 was purchased from the American Type Culture Collection (ATCC, USA). V4 16S ribosomal RNA sequencing was performed to confirm bacterial strains at the species level. The bacteria were grown anaerobically at 37°C for 24 h in brain–heart infusion (BHI) broth (Hopebio, China) supplemented with 5 μg/mL hemin (Hopebio, China) and 0.5 μg/mL vitamin K1 (Hopebio, China). The cultures were centrifuged at 4000 rpm for 15 min and resuspended at a final concentration of 1 × 10^9^ CFU/200 μL in sterile anaerobic phosphate buffer saline (PBS).

### Mouse models and treatments

Male C57BL/6 mice (6–8 weeks old) were purchased from Hunan SJA Laboratory Animal Co. Ltd., China. All animal studies were approved by the Ethics Committee for Animal Experiments of Central South University. All animals were maintained under specific pathogen-free conditions.

Colitis in mice was induced by adding 2% (w/v) DSS (MP Biomedicals) to drinking water for 7 days. All mice were randomly allocated into groups as follows: the control group was administered distilled water and received 200 μL PBS intragastrically once per day for 14 days. The DSS, *B. vulgatus*, 4-Hydroxyproline (4-HP), 3-Aminosalicylic acid (3-ASA), chenodeoxycholic acid (CDCA), and p-hydroxyphenylacetic acid (4-HPAA) groups were administered distilled water for the first 7 days and then fed 2% (w/v) DSS in distilled water from days 8 to 14. In the DSS group, all mice were given 200 μL PBS via oral gavage once per day for 14 days. In the *B. vulgatus* group, all mice were orally administered *B. vulgatus* suspended in sterile anaerobic PBS once daily for 14 days. In the 4-HP, 3-ASA, CDCA, and 4-HPAA groups, the mice were orally administered 4-HP (70 mg/kg), 3-ASA (50 mg/kg), CDCA (70 mg/kg), or 4-HPAA (25 mg/kg) (Macklin Biotech, China) suspended in sterile anaerobic PBS once per day for 14 days. To evaluate the severity of DSS-induced colitis, the body weight and disease activity index (DAI) were recorded daily (Wirtz et al., 2017). After the mice were euthanised, the colon and hippocampal tissues were collected for further investigation. The colon length was measured and colon inflammation was assessed via haematoxylin and eosin staining, as described in a previous study (Wirtz et al., 2017).

Depression-like behaviour in mice was induced by intraperitoneal injection of 5 mg/kg LPS (Sigma-Aldrich, USA) (Arioz et al., 2019). All mice were randomly allocated to the following groups: the control and LPS groups were intragastrically administered PBS for 8 days. On day 7, the LPS group received an intraperitoneal injection of 5 mg/kg LPS, and the control group received an equivalent volume of PBS. The *B. vulgatus*, 3-ASA, CDCA, and 4-HPAA groups were orally administered *B. vulgatus*, 3-ASA, CDCA, and 4-HPAA suspended in sterile anaerobic PBS for 8 days and received an intraperitoneal injection of LPS on day 7. The dose used in this experiment was the same as previously described. As a positive control for the antidepressant effect, in this study, mice were intraperitoneally injected with 10 mg/kg of the tricyclic antidepressant fluoxetine (Macklin Biotech, China) for 7 days. Behavioural analyses were conducted 24 h after LPS injection (described in detail below). After euthanasia, hippocampal tissue was collected for further investigation.

### Histological assessment

1 cm distal colon was fixed with 10% formalin buffer overnight, then embedded in paraffin, cut into 5 μm thick sections, stained with haematoxylin and eosin (HE) and inspection with microscope. A blinded histopathologic analysis was used to evaluate the slides. A 0- to 3-point scale was used to describe the tissue damage (0 = none, 1 = isolated focal epithelial damage, 2 = mucosal erosions and ulcerations, and 3 = extensive damage deep into the bowel wall), the lamina propria inflammatory cell infiltration (0 = infrequent, 1 = increased, some neutrophils, 2 = submucosal presence of inflammatory cell clusters, and 3 = transmural cell infiltrations). Each parameter was calculated and summed to obtain the overall score.

### Behavioural assessment

The open field test (OFT) and tail suspension test (TST) were used to assess depression-like behaviour. All behavioural tests were conducted from 7 a.m. to 12 p.m. Prior to the experiment, the mice were brought to the behavioural room and allowed to rest and habituate for at least 2 h. Blinded experimenters conducted all analyses.

The OFT apparatus comprised 40 × 40 arenas, with the floor divided into 16 equal squares (10 × 10 cm). The centre consisted of four squares, whereas the periphery consisted of 12 squares along the walls. Mice from each group were placed in the centre of the arena and allowed to explore freely. A video camera was mounted on the top of the arena and videotaped for 10 min. The total distance between the arena and its centre area was calculated using Smart Junior software (version 3.0; Panlab, Spain). The TST was conducted in a dimly lit room that was acoustically and visually isolated. Each mouse was individually suspended 50 cm above the floor using adhesive tape placed approximately 1 cm from the tail tip. The immobility time of each mouse was recorded for 6 min using Smart Junior software, and the immobility time was quantified for the last 4 min.

### Transcriptome sequencing and real-time qPCR

RNA preparation, library construction, and sequencing were performed using a DNBSEQ-T7 instrument at the Beijing Genomics Institute. 18,477 genes were analyzed in this study. Gene expression levels were quantified using RSEM software. Gene and genome mapping statistics from RNA-seq analysis are listed in Supplementary Table S4. The differentially expressed genes (DEGs) between samples were analysed using R (version 3.5.0) and the package DESeq2 (version 1.36.0). DEGs were determined using log_2_FoldChange > 1 and adjusted *p*-value <0.05 (Supplementary Table S5). The Kyoto Encyclopedia of Genes and Genomes (KEGG) database was used to extrapolate the differentially expressed pathways. Heatmaps were generated using the R package heatmaps (version 1.20.0). RT-qPCR was performed using a Bio-Rad CFX96 Real-Time PCR platform using the SYBR qPCR Master Mix (Vazyme, China). The primer sequences are listed in Supplementary Table S6.

### Immunofluorescence staining

After transcardial perfusion with ice-cold PBS, the brains were dissected, fixed with 10% formalin buffer overnight, then embedded in paraffin, cut into 20 μm thick sections. Hippocampal tissue sections were blocked in 5% bovine serum albumin for 20 min and then incubated with rabbit anti-BDNF primary antibody (1:200; Proteintech) overnight at 4°C. Subsequently, the sections were washed with 0.01 M PBS and incubated with Cy3 conjugated goat anti-rabbit secondary antibody (1:200; Proteintech) for 1 h at room temperature. The sections were rewashed with 0.01 M PBS and stained with DAPI (2.5 μg/mL, Beyotime Biotechnology, China) for 20 min at room temperature. Anti-claudin-5 (1:500; Invitrogen) and anti-CD31 (1:200; Proteintech) primary antibodies were used to analyse claudin-5 expression in the vascular endothelium of the BBB. CD31 is a vascular endothelial cell marker. Images were captured using an Olympus microscope. The relative fluorescence intensities were calculated using the ImageJ software (version 1.51 K) and the average levels were calculated for each sample.

### Enzyme-linked immunosorbent assay (ELISA)

Total bilateral hippocampi were collected and homogenised in PBS containing protease and phosphatase inhibitors (Beyotime Biotechnology, China). Subsequently, the homogenate was centrifuged (5,000 × *g*, 15 min), and the clarified supernatant was collected for ELISA. BDNF expression levels were measured according to the manufacturer’s protocol (Elabscience, China) and corrected based on the total amount of loaded protein.

### Metabolomics analysis

Faecal, blood, hippocampal samples and bacterial culture supernatant were prepared for metabolomic analysis, according to the manufacturer’s instructions (Beijing Genomics Institute, Beijing, China). Fecal and hippocampal samples should be weighed, added 140ul of 50% water/methanol solution, crushed, and centrifuged to get the supernatant. A total of 350 metabolites were quantified using LC–MS/MS. The analytical instrument for this experiment was LC–MS QTRAP 6500+ (SCIEX). Chromatographic column was BEH C18 (2.1 mm x 10 cm, 1.7um, waters). Ion source was ESI+/ESI–. The R package metaX was used for difference analyses. Principal component analysis was used to visualise differences in the metabolite profiles. Volcano plots were used to illustrate the differences in the quantitative values of the metabolites between the two groups and their statistical significance. Differential metabolites were screened with a ratio ≥ 1.2 or ratio ≤ 0.83 and value of *p* <0.05 (Supplementary Table S7). The KEGG database was used to analyse metabolic pathway enrichment of the differential metabolites. Significantly enriched pathways were identified at *p* < 0.05.

### Measurement of BBB permeability *in vivo*

Evans Blue (EB) tracer dye was used to test BBB permeability as previously described (Bhowmick et al., 2019). EB (2% w/v in sterile PBS) (Sigma-Aldrich) was administered (4 mL/kg) through the tail vein 24 h after LPS treatment. The EB was allowed to circulate for 2 h. After transcardial perfusion with ice-cold PBS, the brains were dissected, weighed, and homogenised in 50% (w/v) trichloroacetic acid (Macklin, China) for 24 h. After centrifugation (2000 × *g* for 15 min) at 4°C, the supernatant was collected. The dye concentration in the supernatant was measured at 620 nm using a spectrophotometer and normalised to the dry weight of brain tissue.

### Transmission electron microscopy

The dissected hippocampi were fixed in 2.5% glutaraldehyde in 0.1 mol/L cacodylic acid buffer (pH 7.3) overnight, and then post-fixed in 1% osmic acid for 2 h. Subsequently, the fixed brains were dehydrated using a graded ethanol series, embedded in an Epon/Araldite mixture, and cut into semi-thin sections. An ultramicrotome (LKB-III, Sweden) was used to cut the slices, which were mounted on copper grids and stained with plumbic nitrate and uranyl acetate. Transmission electron microscopy (Hitachi H-7500, Hitachi, Tokyo, Japan) was used to examine the specimens.

### Statistical analysis

Data are presented as the mean ± standard deviation. Unpaired two-sided *t*-tests were used to compare two experimental groups. A one-way analysis of variance was used to compare three or more experimental groups. The correlation between *B. vulgatus* abundance and PHQ-9 score was measured using the chi-square test. Statistical significance was set at *p* < 0.05. All statistical analyses were performed using Prism 9 software (GraphPad Software, La Jolla, CA, USA).

## Results

### Abundance of *Bacteroides vulgatus* is decreased in patients with IBDD

**Figure 1:**
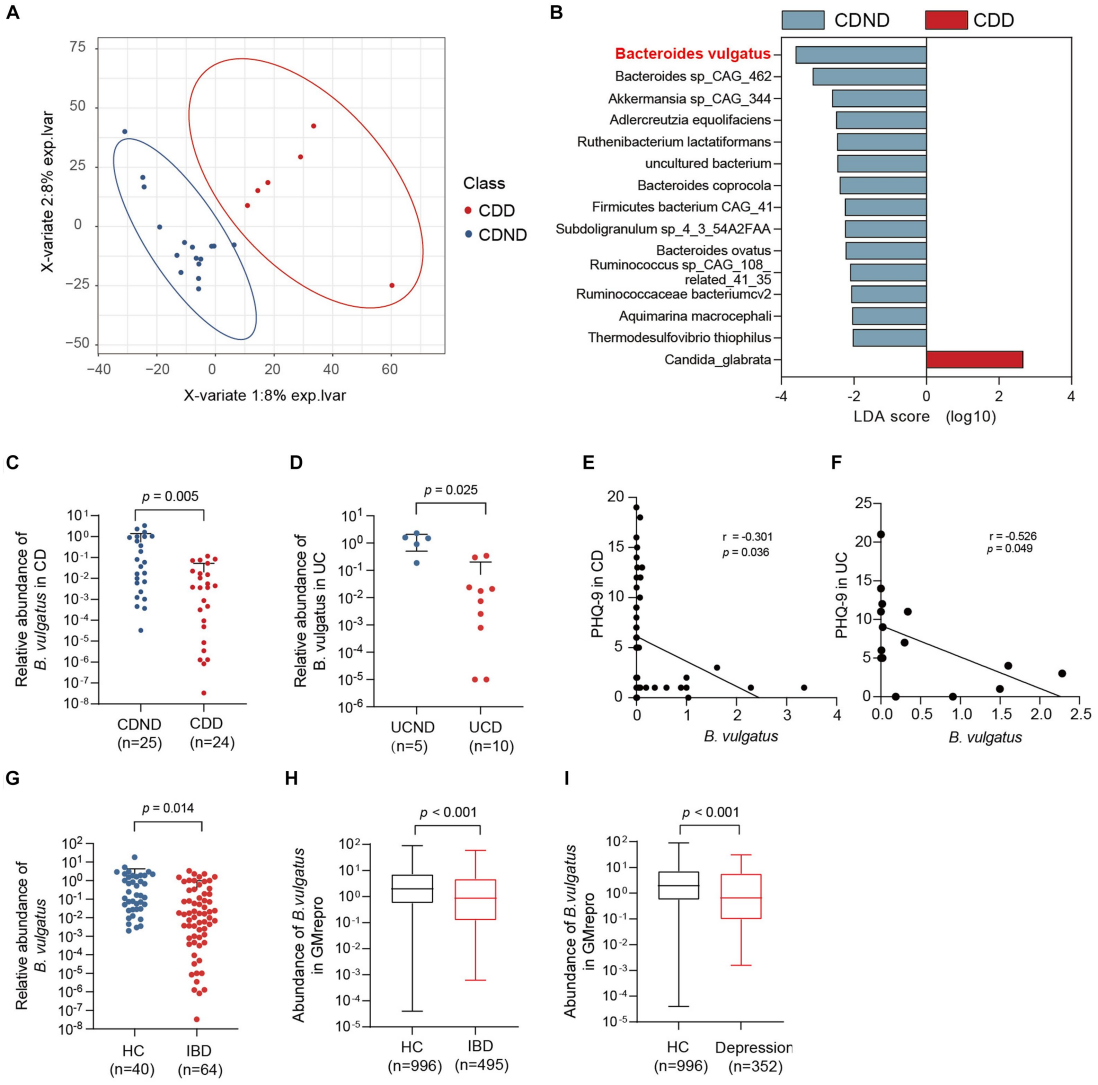
The abundance of *Bacteroides vulgatus* is decreased in patients with IBDD. **(A)** PLS-DA score plot of species abundance in samples from individuals with CDD (*n* = 6, red points) and CDND (*n* = 16, blue points). Permutational multivariate analysis of variance with the Bray–Curtis distance metric was used to assess the significance of differences between the two groups (*p* < 0.001). **(B)** The LEfSe analysis of the bacterial species between CDD and CDND patients. Bacteria species with LDA score greater than 2 were considered to differ between the two groups. Blue bars indicate species enrichment in patients with CDND, and red bars indicate species enrichment in patients with CDD. **(C, D)** The relative abundances of *B. vulgatus* in an CD expanded cohort (*n* = 49) and UC cohort (*n* = 15). The expression of *B. vulgatus* was tested by qPCR of 16S rDNA. **(E)** The correlation of the abundance of *B. vulgatus* in CD feces and PHQ9 score (*n* = 49; two-sided Pearson correlation analysis). **(F)** The correlation of the abundance of *B. vulgatus* in UC feces and PHQ-9 score (*n* = 15; two-sided Pearson correlation analysis). **(G)** The relative abundance of *B. vulgatus* in healthy controls (*n* = 40) and IBD cohort (*n* = 64). **(H, I)** The fecal abundance of *B. vulgatus* in patients with IBD and depression and healthy controls in GMrepo database. CDD, CD patients with depression; CDND, CD patients without depression; IBD, Inflammatory bowel disease; HC, healthy control. Data were expressed as mean ± SD. Differences of data were assessed by unpaired two-sided *t*-test. Exact *p* levels were all provided.

To explore the potential connection between the gut microbiome and CDD, we performed shotgun metagenomic sequencing of faecal samples from 16 patients with CDND and six patients with CDD. First, PLS-DA at the operational taxonomic unit level revealed statistically significant clustering (Figure 1A), suggesting different microbial community structures between the two groups. The LEfSe method was used to identify the specific microbiota communities associated with patients with CDD and CDND. Fifteen discriminative features (LDA > 2, *p* < 0.05) were identified at the species level. *B. vulgatus* abundance was lower in CDD faecal samples than in CDND faecal samples (Figure 1B).

Furthermore, the relative abundance was determined in the expanded cohorts using qPCR of 16S rDNA. We found that patients with CDD had lower *B. vulgatus* levels than those with CDND (Figure 1C). Patients with UCD had lower levels of *B. vulgatus* than patients with UCND (Figure 1D). Moreover, the abundance of *B. vulgatus* in the faeces of patients with CD and UC was negatively correlated with the PHQ-9 score (*r* = −3.01, *p* < 0.05) (Figures 1E,F). Additionally, patients with IBD had a significantly decreased abundance of *B. vulgatus* than that in HCs (Figure 1G).

The GMrepo database was used to determine the abundance of *B. vulgatus* in patients with IBD or depression. We found that patients with IBD had lower *B. vulgatus* levels than HCs (Figure 1H). Moreover, *B. vulgatus* abundance was reduced in patients with depression compared to that in HCs (Figure 1I). Therefore, we speculate that *B. vulgatus* may play an important role in the pathogenesis of IBD and depression.

### *Bacteroides vulgatus* administration ameliorated DSS-induced colitis

**Figure 2:**
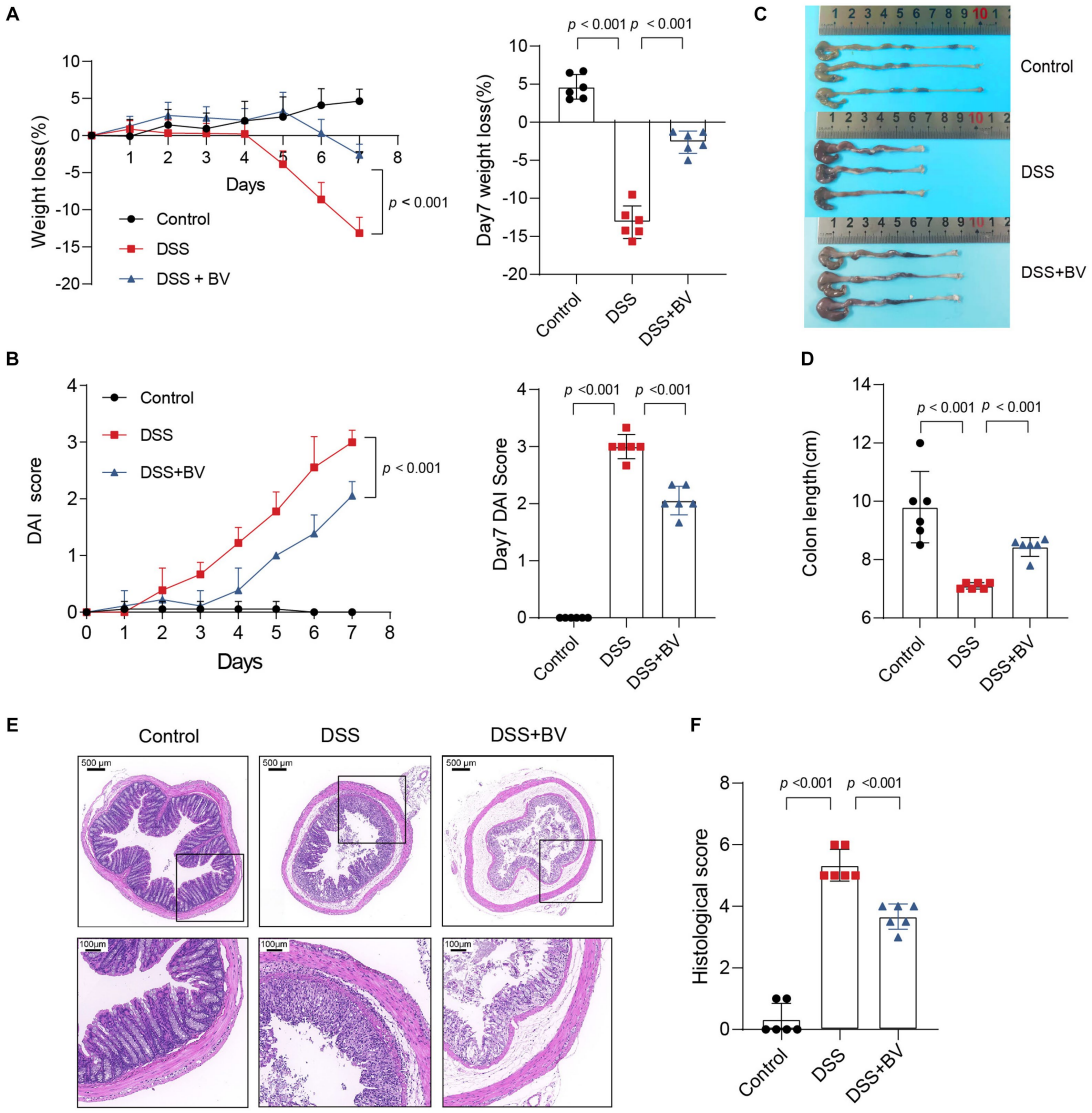
*Bacteroides vulgatus* administration ameliorates DSS-induced colitis. **(A)** Body weight change and statistics of weight loss on day 7. **(B)** DAI score change and statistics of DAI score on day 7. **(C, D)** Representative pictures of colon gross appearance and statistics about colon length. **(E, F)** Representative microscopic pictures of H&E staining (40x and 200x magnification) and statistics of histology score. BV, *B.vulgatus. n* = 6 mice per group. Data were expressed as mean ± SD. Differences of data were assessed by unpaired two-sided *t*-test. Exact *p* levels were all provided.

To further investigate the role of *B. vulgatus* in IBD, we fed C57BL/6 mice 2% DSS with *B. vulgatus* or PBS for 1 week (Supplementary Figure S1A). First, we confirmed that *B. vulgatus* could colonise the gut of mice by comparing the abundance of *B. vulgatus* in the faeces of the *B. vulgatus* and DSS groups (Supplementary Figure S1B). We found that the *B. vulgatus* group showed significantly lower DAI scores, lesser weight loss, and longer colon lengths than the DSS group (Figures 2A–D). Moreover, histological examination of colon sections showed that *B. vulgatus* group mice exhibited less inflammatory cell infiltration, relatively intact colonic architecture, less mucosal damage, and a lower histology score than the DSS group mice (Figures 2E,F). Taken together, these results suggest that *B. vulgatus* protects mice from colitis.

**Figure 3:**
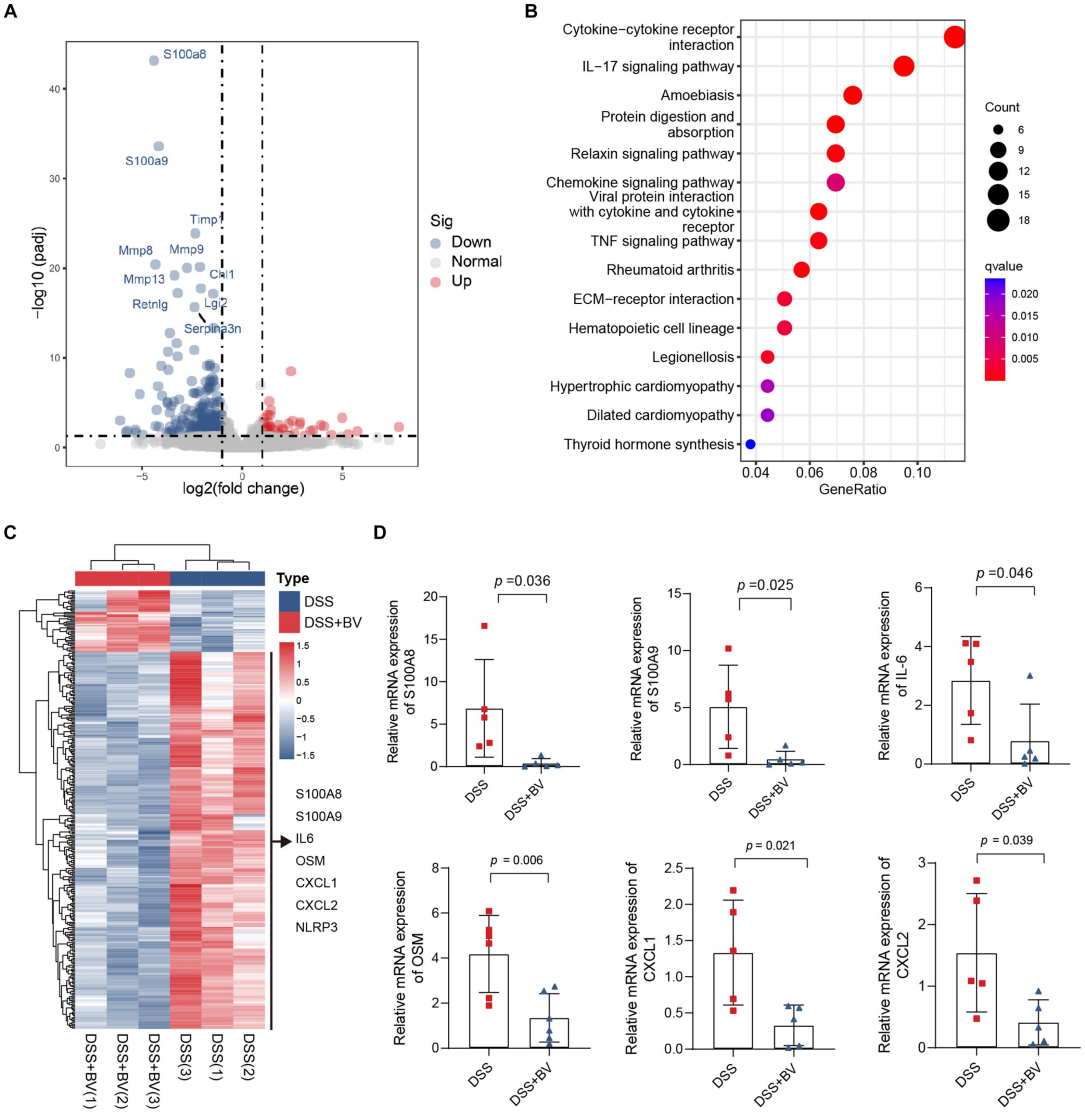
RNA-seq analysis reveals altered gene expression in colon tissue from *Bacteroides vulgatus-*treated mice. **(A)** Volcano map showed the differentially expressed genes (DGEs) caused by *B. vulgatus* in DSS treated mice. The DEGs were determined by using log2FoldChange > 1 and adjusted *p* value <0.05 as criteria (*n* = 3 per group). **(B)** KEGG analysis showed the differentially expressed genes enriched signalling pathway. **(C)** Heatmap analysis revealed the expression of different genes in *B. vulgatus* and DSS groups. **(D)** qPCR was used to determine differential gene expression in DSS and BV colon tissues (*n* = 5 per group). BV, *B. vulgatus.* Data were expressed as mean ± SD. Differences of data were assessed by unpaired two-sided *t*-test. Exact *p* levels were all provided.

Subsequently, to understand why *B. vulgatus* could relieve colitis, we used RNA-seq to analyse altered gene expression in the colon tissue of *B. vulgatus*-treated mice. A volcano map shows the DEGs affected by *B. vulgatus* in DSS-treated mice (Figure 3A). KEGG pathway analysis revealed that the DEGs were mainly enriched in cytokine-cytokine receptor interactions and the IL-17 signalling pathway (Figure 3B). The heatmap shows that IL-6, OSM, CXCL1, and CXCL2 were upregulated in the *B. vulgatus* group compared to their levels in the DSS group (Figure 3C). The expression of these molecules in the colon was confirmed using qPCR analysis (Figure 3D). These results confirm that *B. vulgatus* plays an anti-inflammatory role in colitis.

### *Bacteroides vulgatus* relieves depression-like behaviour in mice

**Figure 4:**
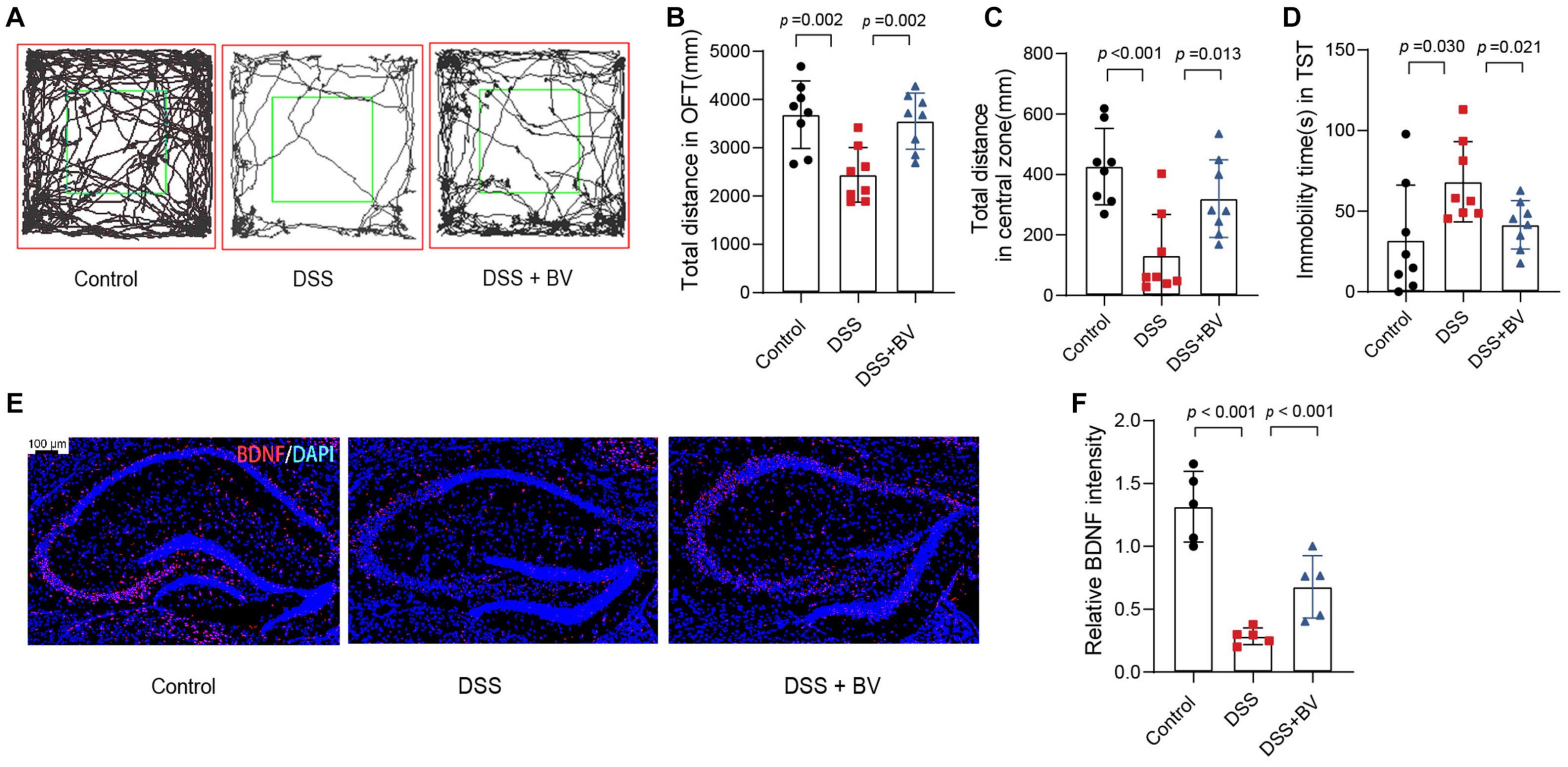
*Bacteroides vulgatus* relieves DSS-induced depression-like behaviour in mice. **(A)** Representative activity tracking of 10 min in the OFT. **(B, C)** Statistical results of the total distance of mice in the open field and central zone. **(D)** TST immobility time statistics. **(E)** Representative images showing BDNF (red) expression in the hippocampus merged with DAPI (blue). **(F)** Statistical results of relative BDNF intensity. Scale bar = 100 μm. BV: *B. vulgatus. n* = 6 mice per group. Data were expressed as mean ± SD. Differences of data were assessed by unpaired two-sided *t*-test. Exact *p* levels were all provided.

Previous studies have reported that DSS-treated mice exhibit depression-like behaviour (Takahashi et al., 2019). In the present study, mice with DSS-induced colitis were used to verify whether *B. vulgatus* relieves the symptoms of depression that accompany IBD. In the OFT, the total distance in the open field and central zone of DSS-treated mice was significantly reduced compared to that of the control mice (Figures 4A–C). In the TST, DSS mice showed a significantly higher duration of immobility than that shown by the control mice (Figure 4D). In contrast, gavage with *B. vulgatus* reversed these changes. Previous studies have indicated that antidepressants increase hippocampal BDNF expression and exert their therapeutic effects (Wang et al., 2008). We examined BDNF expression in the mouse hippocampus. The DSS group had lower hippocampal BDNF levels than the control group. *B. vulgatus* promoted BDNF expression in DSS mice (Figures 4E,F).

Furthermore, we used a mouse model of LPS-induced depression to investigate the antidepressant effects of *B. vulgatus.* The mice in the *B. vulgatus* group showed significantly higher total distances in the open field and central zones than the mice in the DSS group (Supplementary Figures S2A–C)*. B. vulgatus* mice demonstrated significantly shorter immobility durations in the TST than the DSS mice (Supplementary Figure S2D). BDNF levels were decreased in the DSS group but upregulated in the *B. vulgatus* group (Supplementary Figures S2E,F). Taken together, these results suggest that *B. vulgatus* can relieve depression-like behaviour.

### *Bacteroides vulgatus* alters metabolite profile in the stool

**Figure 5:**
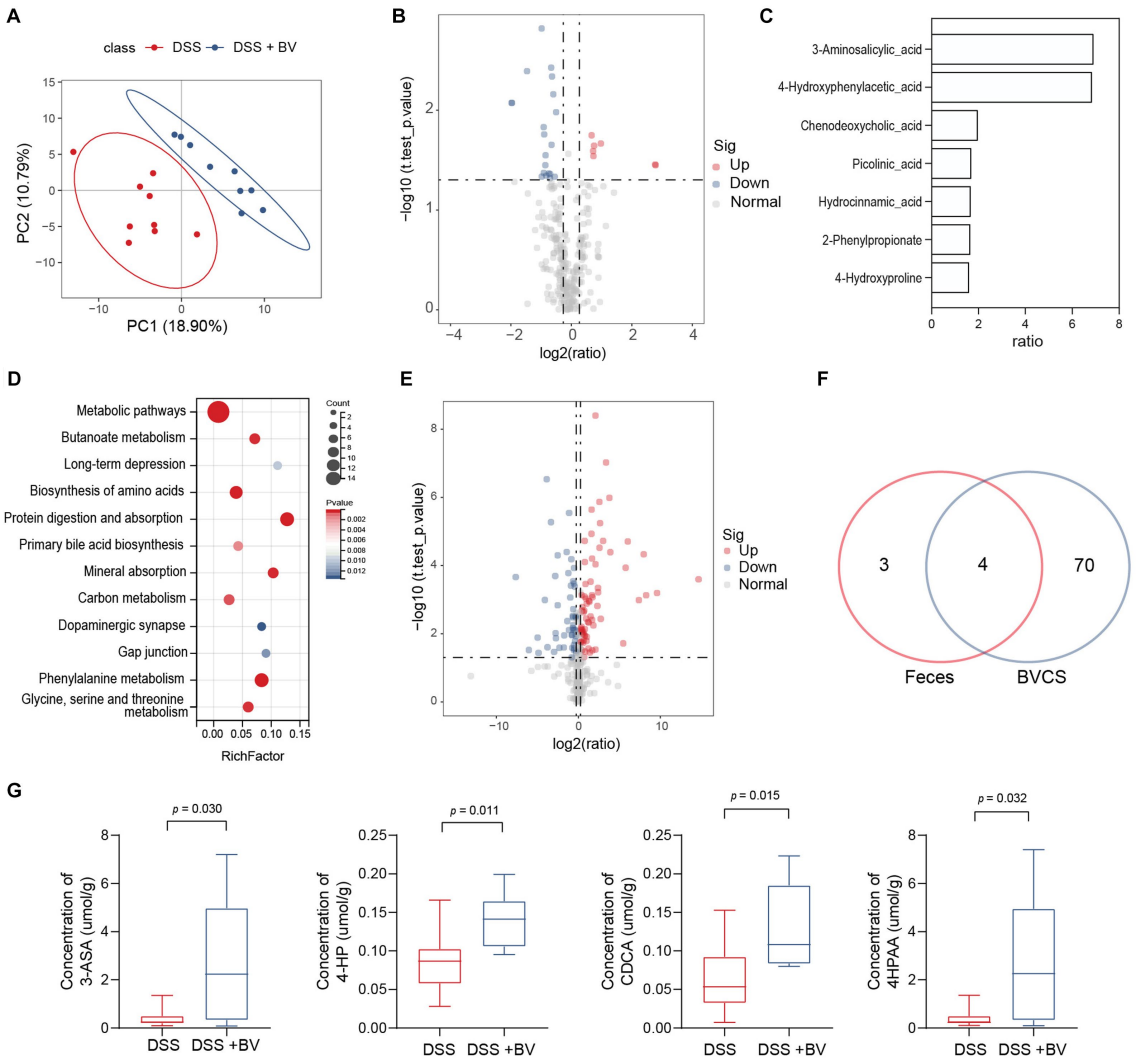
*Bacteroides vulgatus* alters metabolite profile in the stool. **(A)** PLS-DA score plot of metabolites in samples from DSS group (red points) and *B. vulgatus* group (blue points). Permutational multivariate analysis of variance with the Bray–Curtis distance metric was used to assess the significance of differences between the two groups (*p* < 0.001). **(B)** Volcano plots show altered metabolites in feces from DSS mice treated with *B. vulgatus*. **(C)** Up-regulated metabolites attributed to *B. vulgatus* in the feces of DSS-treated mice. **(D)** KEGG analysis showed the *B. vulgatus*-related metabolic pathways. **(E)** Volcano map showed a significant difference between the concentration of metabolites in BVCS and BHI groups. **(F)** Venn diagram shows the number of metabolites in BVCS and the feces of mice affected by *B. vulgatus*. Its intersection represents the common differential metabolites of both group. **(G)** The concentration of common differential metabolites in the *B. vulgatus* group and the DSS group. *n* = 6 mice per group. BV, *B. vulgatus.* Data were expressed as mean ± SD. Differences of data were assessed by unpaired two-sided *t*-test. Exact *p* levels were all provided.

The gut microbiota interacts with the host via metabolites that are produced as intermediates or end products of microbial metabolism. Signals from microbial metabolites influence immune homeostasis and neural activity (Cryan et al., 2019; Krautkramer et al., 2021). Given that disorders in microbial metabolites influence intestinal inflammation and depression-like behaviour, we next sought to determine the effect of *B. vulgatus* on the metabolite profile in the mouse feces. PLS-DA showed an apparent separation of the *B. vulgatus* group from the DSS group (Figure 5A). In total, 27 metabolites were substantially altered in the *B. vulgatus* group compared with those in the DSS group (Figure 5B). Of these, seven metabolites were upregulated (Figure 5C). KEGG pathway analysis revealed metabolic pathways associated with *B. vulgatus*, which included butanoate metabolism, long-term depression, and amino acid biosynthesis (Figure 5D).

To further determine whether the upregulated metabolites were produced by *B. vulgatus,* we performed LC–MS/MS to identify the metabolites in the *B. vulgatus* culture supernatant (BVCS). BHI broth medium for *B. vulgatus* growth was used as the control. The volcano map shows a significant difference between the concentrations of the 125 metabolites in the BVCS and BHI groups (Figure 5E). Moreover, 3-ASA, 4-HP, CDCA, and 4-HPAA levels were significantly higher in the *B. vulgatus* group than in the DSS group and were also highly increased in the BVCS group (Figures 5F,G). Therefore, these four metabolites are produced by *B. vulgatus.* In summary, these results show a significant effect of *B. vulgatus* on the metabolite profile of the mouse feces, as well as the metabolites derived from *B. vulgatus*.

### *Bacteroides vulgatus-*derived metabolites relieve colitis and depression

**Figure 6:**
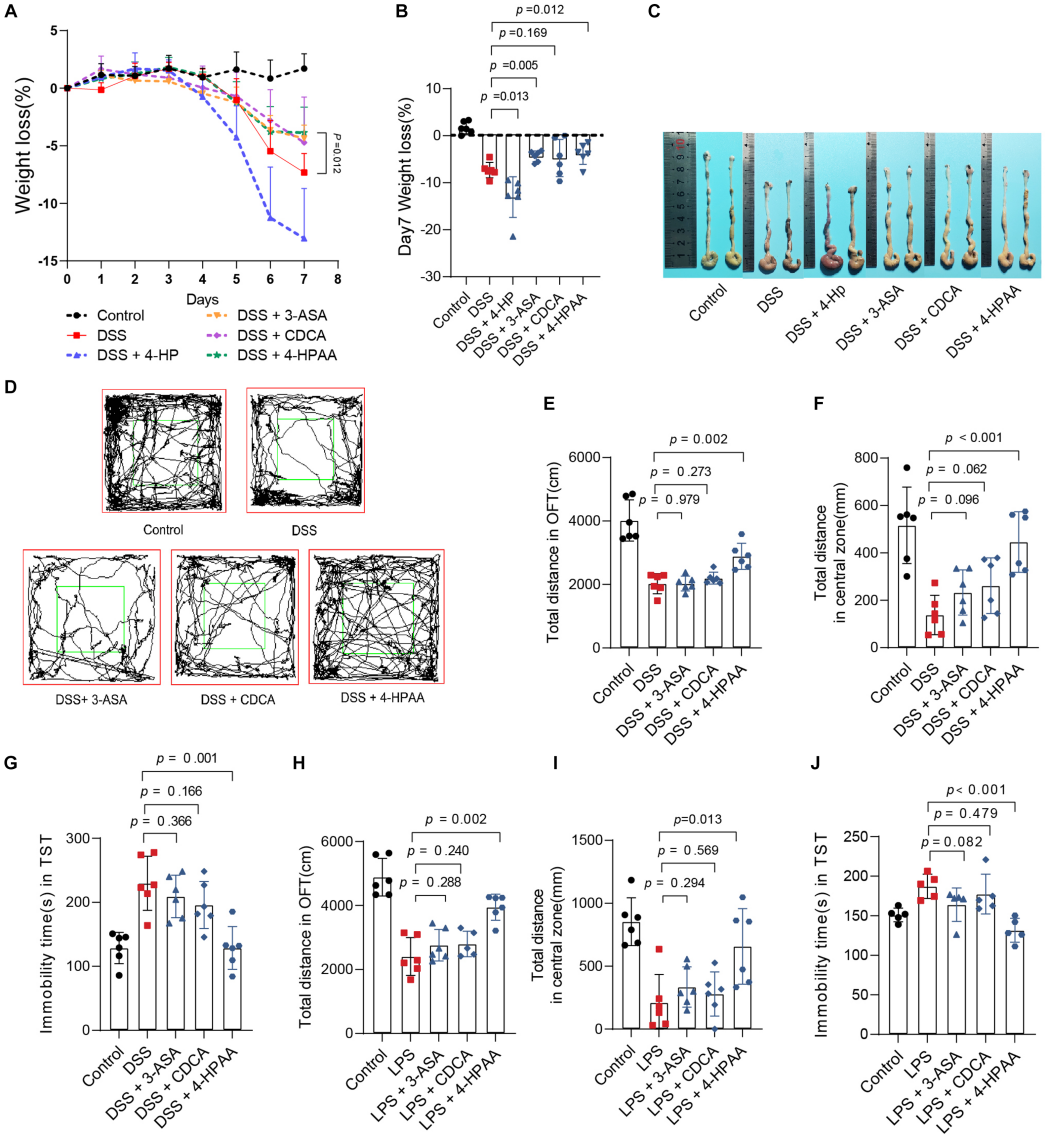
*Bacteroides vulgatus*-derived metabolites relieve colitis and depression. **(A, B)** Body weight change and statistics of weight loss on day 7. **(C)** Representative pictures of colon gross appearance. **(D)** Representative activity tracking of 10 min in the OFT. **(E, F)** Statistical results of the total distance of mice in the open field and central zone. **(G)** TST immobility time statistics. **(H, I)** Statistical results of the total distance of mice in the open field and central zone. **(J)** TST immobility time statistics. *n* = 6 mice per group. Data were expressed as mean ± SD. Differences of data were assessed by unpaired two-sided *t*-test. Exact *p* levels were all provided.

To further identify the role of *B. vulgatus-*derived metabolites in IBD and depression, we orally treated DSS-induced mice with the above four metabolites. The 3-ASA, CDCA, and 4-HPAA groups showed significantly lower DAI scores, longer colon lengths, and lesser weight loss than the DSS group (Figures 6A–C; Supplementary Figures S3A–C). In addition, histological examination of colon sections showed less inflammatory cell infiltration, a relatively intact colonic architecture, less mucosal damage, and a lower histology score in the above-mentioned metabolite-fed mice than in PBS-fed mice (Supplementary Figures S3D,E). However, 4-HP exhibited a contrasting effect. Taken together, these results suggest that 3-ASA, CDCA, and 4-HPAA protected mice from colitis.

Subsequently, we investigated the effects of 3-ASA, CDCA, and 4-HPAA on IBDD. Interestingly, only 4-HPAA alleviated depression-like symptoms in mice with colitis (Figures 6D–G). Additionally, we examined the antidepressant effects of the above three metabolites in a mouse model of LPS-induced depression. These results were similar to those observed in the colitis mice (Figures 6H–J). Moreover, BDNF levels were decreased in the DSS group but were upregulated by *B. vulgatus* and 4-HPAA (Supplementary Figure S3F).

### Role of *Bacteroides vulgatus-*derived 4-HPAA in the gut–brain axis

**Figure 7:**
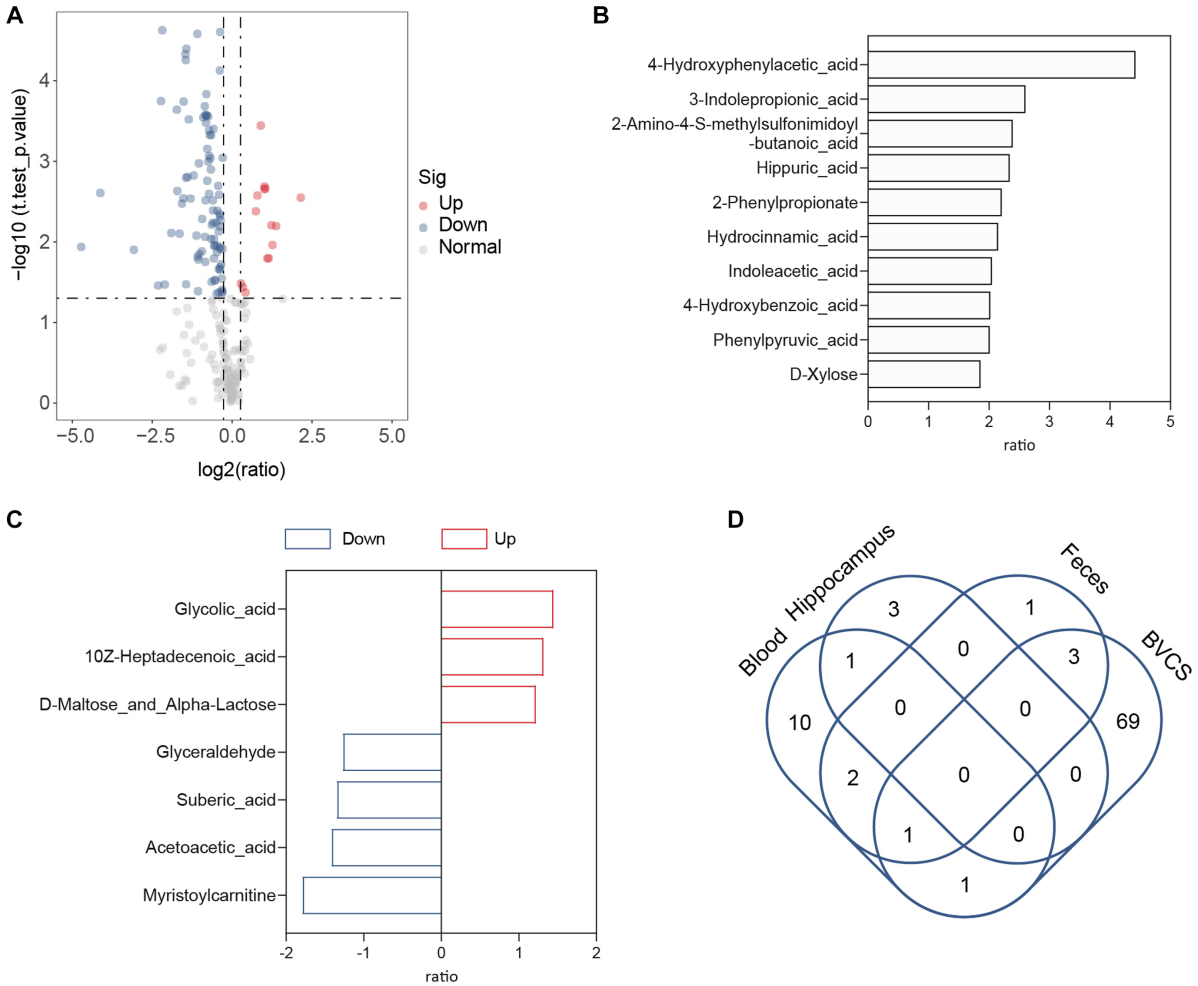
The role of *Bacteroides vulgatus*-derived metabolites in gut-brain axis. **(A)** The volcano map showed a significant difference between the concentration of metabolites in the peripheral blood of mice in the *B. vulgatus* and DSS groups. **(B)** Up-regulated metabolites attributed to *B. vulgatus* in the peripheral blood of DSS-treated mice. **(C)** *B. vulgatus*-related differential metabolites in the hippocampus of colitis mice. **(D)** Venn diagram shows the number of Up-regulated metabolites attributed to *B. vulgatus* in the feces, blood, hippocampus and BVCS. *n* = 6 mice per group.

The gut–brain axis involves enterobacterial metabolites entering the brain through the bloodstream to modulate host behaviour (Morais et al., 2021). An example is the microbial metabolite 4-ethylphenyl sulfate in the intestine, which enters the brain of mice through circulation and alters brain activity and anxiety behaviour (Needham et al., 2022). As depression-like symptoms were relieved after *B. vulgatus* treatment in our previous study using a mouse model, we subsequently investigated whether *B. vulgatus* exerted an antidepressant effect through the gut–brain axis. Metabolites in the peripheral blood and hippocampus of mice were examined in the *B. vulgatus* and DSS groups. Compared to the respective levels in the DSS group, we found that 15 metabolites were increased in the peripheral blood and three metabolites were increased in the hippocampus of the *B. vulgatus* group (Figures 7A–D, Supplementary Table S8). Interestingly, in peripheral blood, 4-HPAA was the most significantly affected by *B. vulgatus*, but no significant differences were observed in the hippocampus. This suggests that *B. vulgatus*-derived 4-HPAA plays an important role in the gut–brain axis and functions in blood vessels rather than in the hippocampus.

### *Bacteroides vulgatus*-derived 4-HPAA regulates the BBB permeability

**Figure 8:**
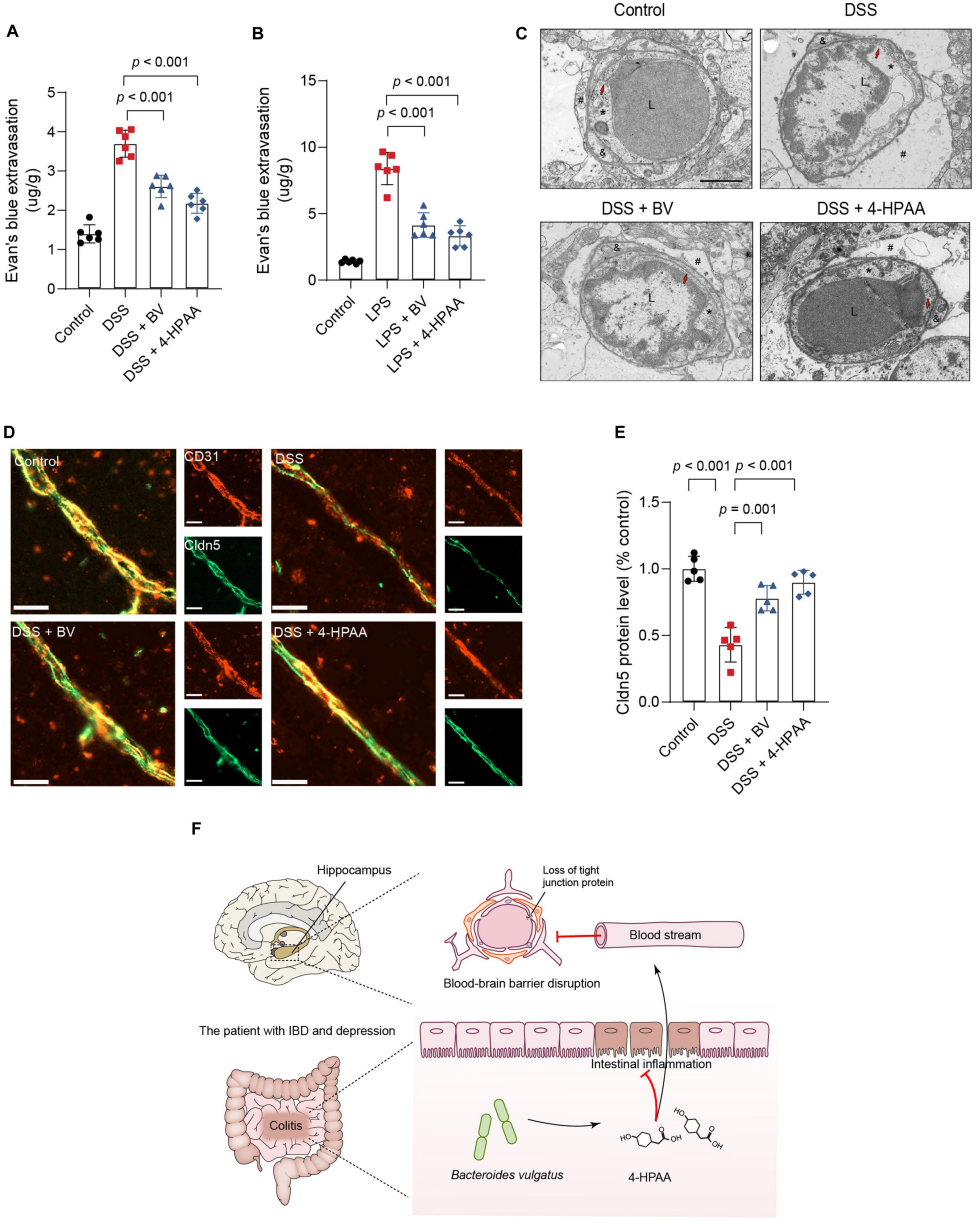
*Bacteroides vulgatus*-derived 4-HPAA regulates BBB permeability. **(A, B)** The BBB’s permeability was tested with Evans blue. *n* = 6 mice per group. **(C)** Representative images of the ultrastructural changes of the BBB tested with Transmission Electron Microscopy. L, lumen of blood vessel; ^*^endothelial cell; ^#^astrocyte; ^&^pericyte; Red arrows indicate the tight junction. Scale bar = 2 μm. **(D)** Representative images showing Claudin-5 (red) expression in the hippocampus BBB vascular endothelium merged with CD31 (blue), a marker for vascular endothelial cells. Scale bar = 20 μm. **(E)** Statistical results of relative Claudin-5 intensity. *n* = 5 mice per group. **(F)** Schematic of hypotheses concerning the mechanisms underlying the role of *B. vulgatus* in regulating IBDD. Cldn5, claudin-5. BV, *B. vulgatus.* Data were expressed as mean ± SD. Differences of data were assessed by unpaired two-sided *t*-test. Exact *p* levels were all provided.

Substances entering the brain are filtered through the BBB. According to previous reports, BBB disruption is a critical factor in depression development (Najjar et al., 2013; Dion-Albert et al., 2022; Wu et al., 2022). Next, we examined changes in BBB permeability in *B. vulgatus*-treated mice using EB tracer dye. DSS and LPS significantly increased intraparenchymal extravasation of EB in mice, which was significantly reduced by 4-HPAA and *B. vulgatus* treatment (Figures 8A,B). These results suggested that *B. vulgatus* and 4-HPAA may have beneficial effects on the BBB. The ultrastructural changes in the hippocampal BBB were examined. Images from the control group showed that the BBB unit was composed of endothelial cells, basal lamina, pericytes, and astrocyte endfeet. In this study, DSS caused changes in the BBB ultrastructure, including a disrupted basement membrane, expanded perivascular space, damaged tight junctions, and swollen astrocyte endfeet. However, these injuries were reversed in the 4-HPAA and *B. vulgatus* groups (Figure 8C).

Previous studies have identified claudin-5 as a key structural component of the tight junctions that maintain BBB integrity (Morais et al., 2021). In this study, immunofluorescence staining was used to analyse claudin-5 expression in the vascular endothelium of the BBB. CD31 is a vascular endothelial cell marker. DSS mice showed significantly reduced expression of claudin-5 in the BBB vascular endothelium compared to that in the control group. However, *B. vulgatus* and 4-HPAA increased claudin-5 expression in DSS-treated mice (Figures 8D,E). Together, these findings indicate that *B. vulgatus* and 4-HPAA protect the BBB permeability in mice by increasing the levels of tight junction proteins.

## Discussion

In the present study, we demonstrated that *B. vulgatus* is a specific microbe associated with IBDD. We also identified the protective effects of *B. vulgatus* against intestinal inflammation using a DSS-induced colitis model. In this study, we present evidence that *B. vulgatus* ameliorates colitis-related depression. Furthermore, our results showed that *B. vulgatus* alleviates depression-like behaviour in mice with colitis through the gut–brain axis mediated by the metabolite 4-HPAA. *B. vulgatus* and 4-HPAA decreased BBB permeability (Figure 8F).

Gut microbiota dysbiosis is closely associated with IBD and depression (Lee and Chang, 2021; Simpson et al., 2021). A previous study demonstrated that the abundance of *Bacteroidaceae* (at the genus level) was lower in UCD than in UCND using 16S rRNA sequencing (Yuan et al., 2021). This suggests that the abundance of *Bacteroidaceae* is reduced in patients with IBD. In our study, we utilised metagenomic analysis to identify the characteristic bacteria of CDD at the species level and identified *B. vulgatus.* Importantly, we used a mouse model of colitis to identify the protective effects of *B. vulgatus* against intestinal inflammation and depression-like behaviour. Therefore, our data further confirm the changes in *Bacteroidaceae* in patients with IBDD. Notably, certain strains of *Bacteroidaceae* (*B. vulgatus*) were identified in this study.

A previous study suggested that *B. vulgatus* increased the number of regulatory/anti-inflammatory CD4^+^ T cell subsets in a mouse model of T cell-induced colitis (Michaelis et al., 2020). Moreover, *B. vulgatus* can ameliorate DSS-induced colitis (Li et al., 2021; Liu et al., 2022). In addition, *B. vulgatus* decreased microbiota dysbiosis and subsequently downregulated NF-kB signalling in the colon, resulting in a decrease in serum TNF-α levels (Yuan and Shen, 2021). In contrast, *B. vulgatus* induced inflammatory cell infiltration in the intestine of an IL10^−/−^ germ-free mouse model (Mills et al., 2022). In this study, all animals were kept under specific pathogen-free conditions rather than germ-free conditions. *B. vulgatus* exhibited beneficial effects similar to those observed in a conventional mouse model. This suggests that *B. vulgatus* plays different roles in common and germ-free mouse models. It is possible that the intestinal microbiota contributes to different functions of *B. vulgatus.*

Patients with IBD commonly receive infliximab to treat intestinal inflammation; however, its efficacy in treating depression remains uncertain (Raison et al., 2013; Horst et al., 2015; Thillard et al., 2020). In this study, we found that three *B. vulgatus*-derived metabolites (3-ASA, CDCA, 4-HPAA) had anti-inflammatory effects, whereas only 4-HPAA had antidepressant effects. This demonstrates that depressive symptoms are not completely alleviated by anti-inflammatory treatment alone. Additional antidepressants, such as tricyclic antidepressants and selective serotonin reuptake inhibitors are often used to treat depressive symptoms in patients with IBD (Frolkis et al., 2019; Mikocka-Walus et al., 2020). However, the combination of biologics and antidepressants increases the risk and economic burden on patients. Moreover, no effective regimen has been developed to treat intestinal inflammation and depression simultaneously. In our study, we found that *B. vulgatus* and *B. vulgatus-*derived 4-HPAA had dual effects in relieving intestinal inflammation and depressive symptoms in mice with colitis. Therefore, administration of *B. vulgatus* or 4-HPAA supplementation is a promising therapeutic strategy for treating patients with IBD, especially those with depression.

The gut–brain axis is considered a key regulator of neural function (Cryan et al., 2019). *Lactiplantibacillus plantarum* DMDL 9010 and *Enterococcus faecalis* 2001 have been reported to alleviate DSS-induced colitis and behavioural disorders through the gut–brain axis (Takahashi et al., 2019; Huang et al., 2022). However, few studies have explored how intestinal bacteria regulate the gut–brain axis through bacteria-derived metabolites in depression. We found that *B. vulgatus* regulates BDNF expression in the hippocampus, suggesting that it exerts antidepressant effects through the gut–brain axis. Furthermore, we identified key *B. vulgatus* metabolites through metabolome analysis and confirmed that the metabolite 4-HPAA directly regulates the gut–brain axis by protecting BBB permeability. Importantly, we identified the metabolites in BVCS and confirmed that 4-HPAA is indeed a metabolite produced by *B. vulgatus* rather than a dietary source.

Clinical and experimental evidences suggest that BBB hyperpermeability contributes to depression (Najjar et al., 2013; Wu et al., 2022). Additionally, the integrity of the BBB can be enhanced by microbe-derived metabolites, such as short-chain fatty acids and trimethylamine N-oxide (Hoyles et al., 2018, 2021). These results suggest a close relationship among bacteria-derived metabolites, BBB integrity, and depression. In our study, *B. vulgatus*-derived 4-HPAA protected BBB integrity. This is similar to a previous report showing that 4-HPAA reduces rat lung microvascular endothelial cell monolayer permeability (Liu et al., 2014). Claudin-5 is the most enriched tight junction protein in the BBB and its dysfunction is implicated in depression (Greene et al., 2019; Morais et al., 2021). Notably, BBB disruption can result in the loss of BDNF in the brain (Lesniak et al., 2021). In our study, *B. vulgatus* and 4-HPAA increased claudin-5 expression in the BBB vascular endothelium. Therefore, we speculated that *B. vulgatus* and 4-HPAA reduced BBB permeability via claudin-5. Moreover, *B. vulgatus* may regulate the BBB permeability and affect BDNF secretion in the brain. Nevertheless, given the differences between humans and experimental animals, further research is needed to investigate whether *B. vulgatus* and 4-HPAA can achieve similar therapeutic effects in humans.

## Conclusion

In summary, *B. vulgatus* and *B. vulgatus*-derived 4-HPAA can relieve intestinal inflammation and alleviate depression-like behaviour in a mouse model. By increasing the expression of the tight junction protein claudin-5 in the vascular endothelium of the BBB, *B. vulgatus* and 4-HPAA play critical roles in gut–brain communication. Our research suggests that the administration of *B. vulgatus* or 4-HPAA supplementation may be a promising therapeutic strategy for treating IBD, especially IBDD.

## Data availability statement

The datasets presented in this study can be found in online repositories. The names of the repository/repositories and accession number(s) can be found at: https://figshare.com/, 10.6084/m9.figshare.22549636.v2. and https://www.ncbi.nlm.nih.gov/geo/, GSE246118.

## Ethics statement

The studies involving humans were approved by The Institutional Review Board of the Third Xiangya Hospital of Central South University. The studies were conducted in accordance with the local legislation and institutional requirements. The participants provided their written informed consent to participate in this study. The animal study was approved by The Animal Experimental Ethics Committee of Central South University. The study was conducted in accordance with the local legislation and institutional requirements.

## Author contributions

XWu: Conceptualization, Data curation, Formal analysis, Investigation, Methodology, Project administration, Software, Supervision, Validation, Visualization, Writing – original draft, Writing – review & editing, Funding acquisition. JX: Conceptualization, Data curation, Investigation, Methodology, Software, Supervision, Validation, Visualization, Writing – review & editing. JL: Methodology, Project administration, Supervision, Writing – review & editing. MD: Methodology, Project administration, Supervision, Writing – review & editing. ZS: Methodology, Writing – review & editing. KN: Methodology, Writing – review & editing. WL: Investigation, Methodology, Writing – review & editing. CZ: Investigation, Methodology, Software, Writing – review & editing. KM: Investigation, Methodology, Writing – review & editing. XC: Investigation, Methodology, Writing – review & editing. XWa: Conceptualization, Data curation, Funding acquisition, Investigation, Project administration, Resources, Supervision, Writing – review & editing.

## Footnotes

^1^ https://gmrepo.humangut.info/home

## Supplementary material

Supplementary FIGURE S1 Experimental design for DSS-induced colitis. **(A)** Experimental design for DSS-induced colitis. **(B)** An analysis of the relative abundance of *B. vulgatus * in mice’ stools after the last day of DSS treatment. Data were expressed as mean ± SD. Differences of data were assessed by unpaired two-sided t-test. Exact *p* levels were all provided.

Supplementary FIGURE S2 *B. vulgatus * relieves LPS-induced depression-like behaviour in mice. **(A)** Representative activity tracking of 10 min in the OFT. **(B, C)** Statistical results of the total distance of mice in the open field and central zone. **(D)** TST immobility time statistics. **(E)** Representative images showing BDNF expression in hippocampus merged with DAPI. Scale bar = 100 μm. **(F)** Statistical results of relative BDNF intensity. n = 6 mice per group. FLX: Fluoxetine. Data were expressed as mean ± SD. Differences of data were assessed by unpaired two-sided t-test. Exact *p* levels were all provided.

Supplementary FIGURE S3 *B. vulgatus *-derived metabolites relieve colitis and depression. **(A, B)** DAI score change and statistics of DAI score on day 7. **(C)** Statistics of colon length. **(D, E)** Representative microscopic pictures of H&E staining (200x magnification) and statistics of histology score. **(F)** Expression of BDNF in the hippocampus was analyzed by ELISA. n = 6 mice per group. Data were expressed as mean ± SD. Differences of data were assessed by unpaired two-sided t-test. Exact *p* levels were all provided.

## Glossary

IBD: Inflammatory bowel diseases
UC: Ulcerative colitis
CD: Crohn’s disease
IBDD: IBD patients with depression
IBDND: IBD patients without depression
CDD: CD patients with depression
CDND: CD patients without depression
UCD: UC patients with depression
UCND: UC patients without depression
HC: Healthy controls
OFT: Open field test
TST: Tail suspension test
B. vulgatus: Bacteroides vulgatus
DSS: Dextran sulfate sodium salt
DAI: Disease activity index
BBB: Blood–brain barrier
BDNF: Brain-derived neurotrophic factor
PHQ-9: Patient Health Questionnaire-9
