# Supplementary figures and images for: Bacteroides vulgatus alleviates dextran sodium sulfate-induced colitis and depression-like behaviour by facilitating gut-brain axis balance
Source: Front Microbiol. 2023 Nov 16;14:1287271. doi: 10.3389/fmicb.2023.1287271 (PMC10687441; doi:10.3389/fmicb.2023.1287271)

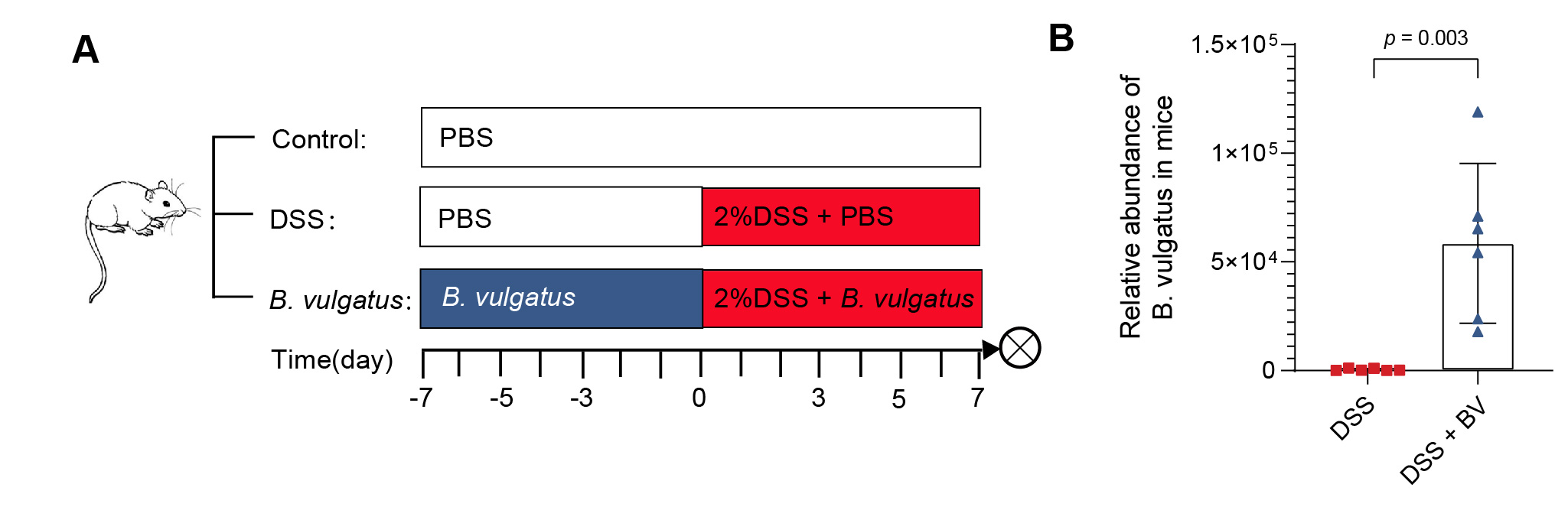

Supplement: Supplementary FIGURE S1 — Experimental design for DSS-induced colitis. (A) Experimental design for DSS-induced colitis. (B) An analysis of the relative abundance of B. vulgatus in mice’ stools after the last day of DSS treatment. Data were expressed as mean ± SD. Differences of data were assessed by unpaired two-sided t-test. Exact p levels were all provided. [file Image_1.JPEG]

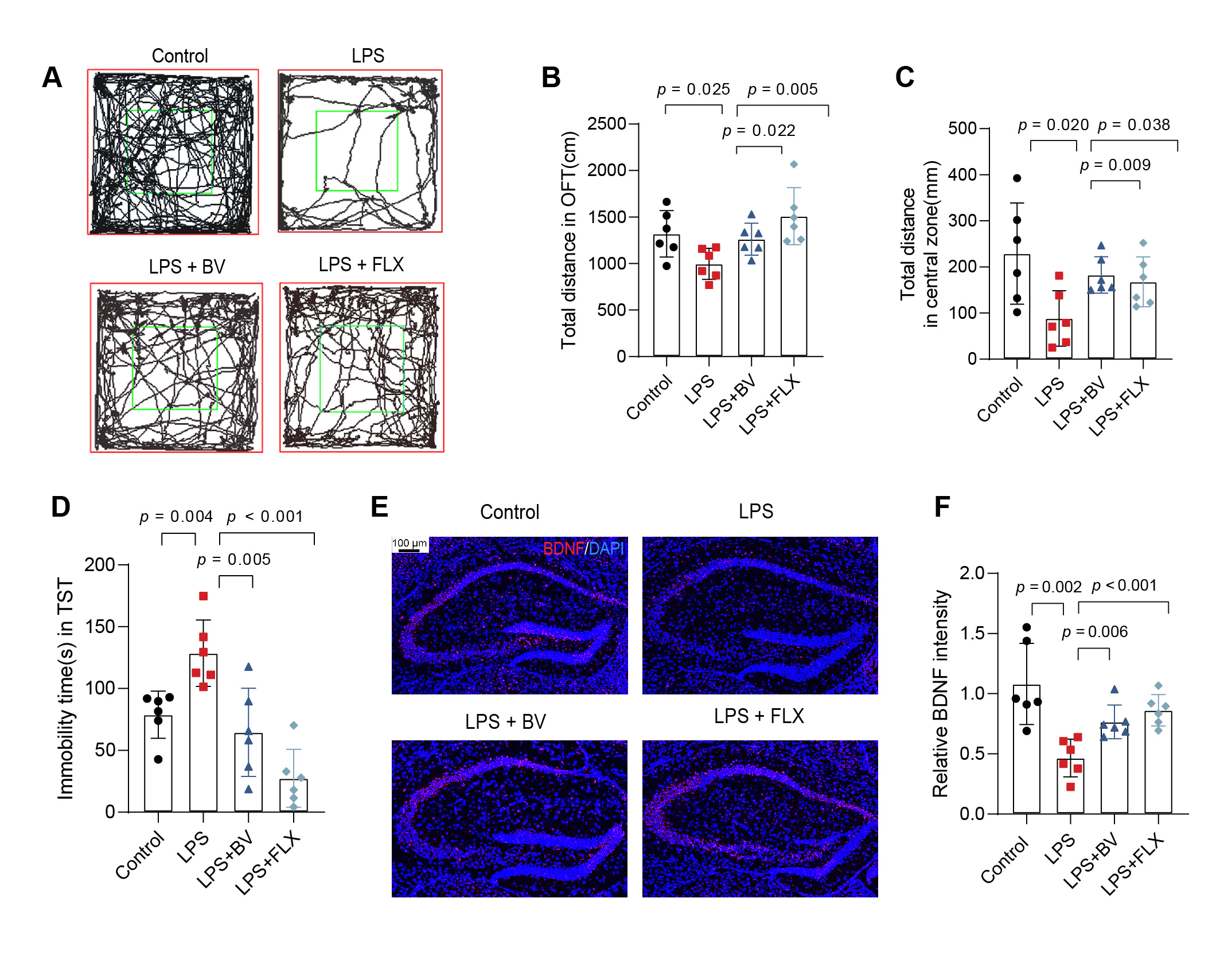

Supplement: Supplementary FIGURE S2 — B. vulgatus relieves LPS-induced depression-like behaviour in mice. (A) Representative activity tracking of 10 min in the OFT. (B, C) Statistical results of the total distance of mice in the open field and central zone. (D) TST immobility time statistics. (E) Representative images showing BDNF expression in hippocampus merged with DAPI. Scale bar = 100 μm. (F) Statistical results of relative BDNF intensity. n = 6 mice per group. FLX: Fluoxetine. Data were expressed as mean ± SD. Differences of data were assessed by unpaired two-sided t-test. Exact p levels were all provided. [file Image_2.JPEG]

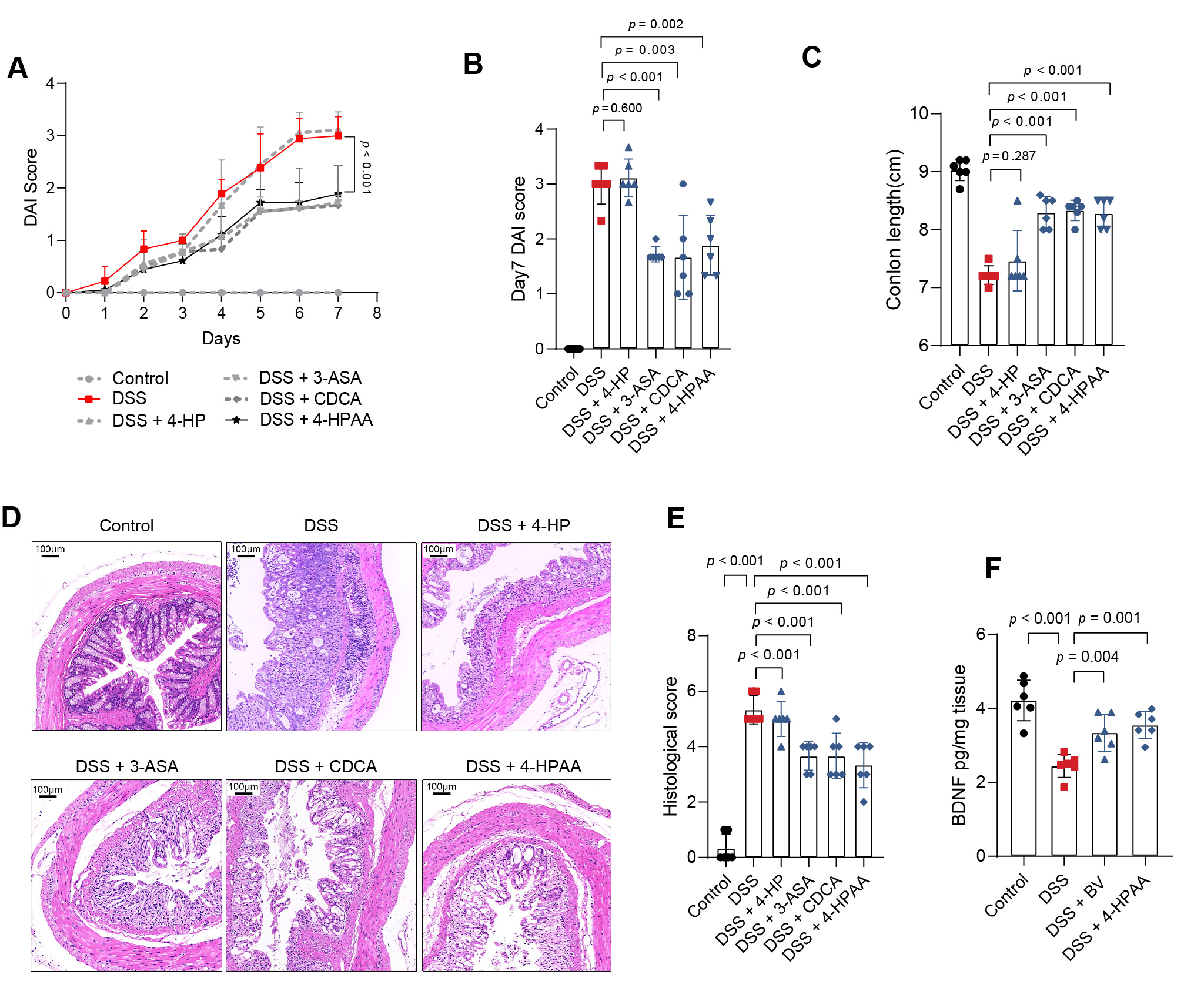

Supplement: Supplementary FIGURE S3 — B. vulgatus -derived metabolites relieve colitis and depression. (A, B) DAI score change and statistics of DAI score on day 7. (C) Statistics of colon length. (D, E) Representative microscopic pictures of H&E staining (200x magnification) and statistics of histology score. (F) Expression of BDNF in the hippocampus was analyzed by ELISA. n = 6 mice per group. Data were expressed as mean ± SD. Differences of data were assessed by unpaired two-sided t-test. Exact p levels were all provided. [file Image_3.JPEG]
